# Characterization of Novel Biopolymer Blend Mycocel from Plant Cellulose and Fungal Fibers

**DOI:** 10.3390/polym13071086

**Published:** 2021-03-30

**Authors:** Ilze Irbe, Inese Filipova, Marite Skute, Anna Zajakina, Karina Spunde, Talis Juhna

**Affiliations:** 1 Latvian State Institute of Wood Chemistry, Dzerbenes 27, LV-1006 Riga, Latvia; inese.filipova@inbox.lv (I.F.); polarlapsa@inbox.lv (M.S.); 2 Latvian Biomedical Research and Study Centre, Ratsupites 1 k. 1, LV-1067 Riga, Latvia; karina.spunde@biomed.lu.lv; 3 Water Research and Environmental Biotechnology Laboratory, Riga Technical University, P. Valdena 1-303, LV-1048 Riga, Latvia; talis.juhna@rtu.lv

**Keywords:** air permeability, fungal fibers, hemp fibers, microstructure, mechanical properties, mycocel, softwood fibers, virus membrane filtration

## Abstract

In this study unique blended biopolymer mycocel from naturally derived biomass was developed. Softwood Kraft (KF) or hemp (HF) cellulose fibers were mixed with fungal fibers (FF) in different ratios and the obtained materials were characterized regarding microstructure, air permeability, mechanical properties, and virus filtration efficiency. The fibers from screened Basidiomycota fungi *Ganoderma applanatum* (Ga), *Fomes fomentarius* (Ff), *Agaricus bisporus* (Ab), and *Trametes versicolor* (Tv) were applicable for blending with cellulose fibers. Fungi with trimitic hyphal system (Ga, Ff) in combinations with KF formed a microporous membrane with increased air permeability (>8820 mL/min) and limited mechanical strength (tensile index 9–14 Nm/g). HF combination with trimitic fungal hyphae formed a dense fibrillary net with low air permeability (77–115 mL/min) and higher strength 31–36 Nm/g. The hyphal bundles of monomitic fibers of Tv mycelium and Ab stipes made a tight structure with KF with increased strength (26–43 Nm/g) and limited air permeability (14–1630 mL/min). The blends KF FF (Ga) and KF FF (Tv) revealed relatively high virus filtration capacity: the log_10_ virus titer reduction values (LRV) corresponded to 4.54 LRV and 2.12 LRV, respectively. Mycocel biopolymers are biodegradable and have potential to be used in water microfiltration, food packaging, and virus filtration membranes.

## 1. Introduction

The importance of biobased polymers is well known, and much research and development activities concern the use of biobased polymers in science, engineering, and industry. Biopolymers from renewable resources are used in multiple fields, namely health, food, energy, and the environment, due to their intrinsic features, versatility, biocompatibility, and degradability. Besides, the widespread use of biopolymers also addresses concerns about environmental sustainability [1]. As long as biopolymers stand as a sustainable, biodegradable compound, new biopolymer products and materials filled, blended, or reinforced with natural fibers, will predominate with the promise of sustainability benefits [2].

Generally, biobased polymers are classified into three classes: (1) naturally derived biomass polymers such as cellulose, cellulose acetate, starches, chitin, modified starch, etc.; (2) bio-engineered polymers bio-synthesized by using microorganisms and plants such as poly(hydroxy alkanoates (PHAs), poly(glutamic acid), etc.; (3) synthetic polymers produced from naturally derived molecules or by the breakdown of naturally derived macromolecules through the combination of chemical and biochemical processes such as polylactide (PLA), poly(butylene succinate) (PBS), bio-polyolefins, bio-poly(ethylene terephtalic acid) (bio-PET) [3]. The first and second class polymers are biodegradable, they allow for more efficient production, which can produce desired functionalities and physical properties, but chemical structure designs have limited flexibility. The third class polymers such as bio-polyolefins and bio-PET are not biodegradable and the only contribution for reducing environmental impact comes from reducing the carbon footprint. The origin of a polymer does not determine its biodegradability; this condition depends on the chemical structure of the polymer [3].

The naturally derived biopolymers, among others, include polysaccharides of plant and fungal origin. Polysaccharides are nontoxic and biodegradable, which increases their potential application in biopolymers. The most used biopolysaccharides are obtained from plant origin (e.g., cellulose), microbial origin (e.g., bacterial cellulose), and animal origin (e.g., chitin/chitosan). Moreover, these natural derivatives present a considerable number of reactive functional groups (e.g., hydroxyl, carboxyl, and amino groups), which significantly increase their applicability through chemical modification or physical blend [1]. Cellulose is the most abundant polysaccharide of natural origin in the world, and is mostly produced by plants. The cellulose chains structure leads to areas of high crystallinity within the polymer and to high stability structures, which as a consequence promote considerable strength, remarkable inertness, and insolubility in water and common organic solvents [4]. In its turn, fungal cell walls share a common chemical structure composed of homo- and heteropolysaccharides, protein, protein–polysaccharide complexes, lipids, melanin, and polysaccharide chains of chitin. Chitin is a biopolymer of N-acetylglucosamine with some glucosamine, which is the main component of the cell walls of fungi and is considered the second most abundant natural polymer after cellulose [5].

Current research in membrane science is now focusing more on biopolymers from natural raw materials with a well-defined structure to develop new membrane materials. In fact, the combination of polysaccharides and proteins is a method frequently used to design blended materials with improved performance regarding swelling, mechanical resistance, and biocompatibility, among other features. The attention has been focused also on membranes based on chitosan blended with other biomacromolecules such as alginate, cellulose, collagen, gelatin, keratin, sericin, and soy protein [6]. Chitin-related materials from fungal sources with focus on nanocomposites and nanopapers have been suggested as greener alternative to synthetic polymers [7]. Fungal chitin–glucan nanopapers have manufactured from chitin nanofibrils, which is a native composite material (chitin–glucan) combining the strength of chitin and the toughness of glucan. These nanopapers showed distinct physico-chemical surface properties, being more hydrophobic than crustacean chitin [8]. Nanopapers from chitin nanofibrils have exhibited tunable mechanical and surface properties with potential use in coatings, membranes, packaging [9] and ultrafiltration of organic solvents and water [10]. Fungal mycelium–nanocellulose has been produced by the agitated liquid culture of a white-rot fungus with nanocellulose as part of the culture media. The obtained biomaterials are suggested for diverse applications, including packaging, filtration, and hygiene products [11].

The literature survey demonstrates that fungal chitin nanofibrils have been used to manufacture nanopapers. Additionally, fungal mycelium has been combined with nanocellulose to obtain a blended biomaterial. However, there are no studies on blended biopolymers from plant cellulose and fungal hyphae. The aim of this study was to develop a novel blended biopolymer from naturally derived biomass that is made of softwood cellulose fibers and fungal fibers (hyphae) and to test it for air permeability, mechanical properties, and virus filtration efficiency. More detailed investigation of specific properties of biomaterial blend containing fungal fibers from basidiomycete *Ganoderma applanatum* is published in our previous paper [12]. The cellulose fibers from softwood and hemp shives are easily available natural resource. The basidiomycetes selected for this study have shown a high importance in medical and nutritional applications. For example, *Ganoderma applanatum* has been reported for its phytochemical properties for potential application in nanotechological engineering for clinical use [13]. *Fomes fomentarius* has been used in a traditional medicine for centuries and is still economically important as a source of medicinal and neutraceutical products [14]. *Trametes versicolor* is known for its general health-promoting effects and is widely employed in traditional medicine [15]. *Agaricus bisporus* represents the leading position among edible cultivated mushrooms [16].

## 2. Materials and Methods

### 2.1. Synthesis of Biopolymers

The screening of several basidiomycetes was carried out to select the fungal candidates forming homogenous hyphal biomass convenient for biopolymer material development. The fungal biomass was obtained from (1) fruiting bodies of the forest growing species *Ganoderma applanatum* (Pers.) Pat., *Fomes fomentarius* (L.) Fr., *Lentinus lepideus* (Fr.) Fr., *Polyporus squamosus* (Huds.) Fr., *Fomitopsis betulina* (Bull.) B.K. Cui, (2) commercially cultivated mushroom stipes of *Agaricus bisporus* (J.E. Lange) Imbach and (3) mycelium of pure culture strain *Trametes versicolor* CTB 863 A.

Fungal biomass was kept in 4% NaOH solution for 24 h at a room temperature in order to extract proteins and alkali soluble polysaccharides. Then the samples were washed in tap water and mechanically disintegrated using Blendtec 725 (Orem, UT, USA) at 360 W for 30 s. Obtained fungal fibers (FF) were dried at room temperature and kept in a dry state until used.

Bleached softwood Kraft fibers (KF) were provided by Metsä Fibre (Äänekoski, Finland) as pressed sheets and used without specific pre-treatment.

Hemp fibers (HF) were obtained from industrial hemp *Cannabis sativa* (USO-31). After the decortication fibers were treated in 4% NaOH solution at 165 °C for 75 min, then washed with tap water to neutral and refined using Blendtec 725 (Orem, UT, USA) at 179 W for 7 min at 1.5% consistency, dried at room temperature and kept in dry state until used.

For biopolymer blend development, a certain amount (g) of FF in combinations with KF and HF fiber pulp in different mass ratios (50:50 and 33:33:33) was placed in a glass baker and soaked in 1–2 L of distilled water for 8 h, then disintegrated using 75,000 revolutions in the disintegrator (Frank PTI, Laakirchen, Austria). Material sheets were produced according to ISO 5269-2:2004 with a Rapid Köthen paper machine (Frank PTI, Laakirchen, Austria). Grammage (weight per unit area in g/m^2^) of samples was calculated by dividing the mass with area according to ISO 536:2019. At least five parallel samples at grammage 50 ± 15 g/m^2^ of each composition were prepared.

### 2.2. Micromorphology

The micromorphology was examined by a light microscope (LM) Leica DMLB (Leica Microsystems GmbH, Wetzlar, Germany) at a magnification of 200×. Lactophenol blue solution (Fluka) was used to observe cyanophilic reaction of fungal hyphae. The images were captured by a video camera Leica DFC490 using calibrated image analysis software Image-Pro plus 6.3 (Media Cybernetics, Inc., Rockville, MD, USA).

For scanning electron microscopy (SEM), the surface of samples was coated with gold plasma using a K550X sputter coater (Emitech, Ashford, UK) and examined with Vega TC (Tescan, Brno-Kohoutovice, Czech Republic) with accelerating voltage of 15 kV, software 2.9.9.21.

### 2.3. Air Permeability

Air permeability was tested by Bendsen method according to ISO 5636-3:2013 using an air permeability tester 266 (Lorentzen and Wettre, Stockholm, Sweden). Samples were clamped between a metal ring and a rubber gasket and the air flow rate was measured through sample area of 10 cm^2^ under 1.47 kPa air pressure for 5 s. Commercially available disposable face masks consisting of three layers made of polypropylene spunbond-meltblown-spunbond nonwoven fabrics were tested for air permeability as comparison for developed biopolymer samples.

### 2.4. Mechanical Properties

For evaluation of tensile properties samples with a width of 1 cm were prepared with a strip cutter (Frank-PTI, Laakirchen, Austria) and tested according to ISO 1924-1:1992 using a tensile tester vertical F81838 (Frank-PTI, Laakirchen, Austria). Tensile index (Nm/g) was calculated by dividing measured maximum tensile strength (N/m) by grammage (g/m^2^) of test sample. Breaking length (km) and stretch (%) was used as calculated by software of equipment. Burst index (kPa m^2^/g) was calculated dividing the measured burst strength (kPa) by grammage (g/m^2^), where burst strength was measured as hydrostatic pressure necessary to cause rupture in a circular area of a 4 cm diameter of sample according to ISO 2758:2014 using burst tester (Frank-PTI, Laakirchen, Austria).

### 2.5. Virus Preparation and Membrane Filtration Procedure

The recombinant Semliki forest virus (SFV) pSFVenh/Luc, encoding firefly luciferase gene, was produced as previously described [17,18]. The virus-containing cell medium was harvested and concentrated by ultracentrifugation through two sucrose cushions, as previously described [19]. The virus was rapidly frozen and subsequently used as a virus stock. The virus titre expressed in infectious units per ml (i.u./mL) was quantified by immunostaining with rabbit polyclonal antibodies specific to the nsp1 subunit of SFV replicase, as previously described [20].

The ability of developed biopolymer materials to retain/remove virus particles was tested using centrifugal filtration test. EMA/CPMP/BWP/268 guidelines were considered for development of virus filtration procedures and calculation of virus reduction values. Two-layer cellulose hygienic paper (AB Grigeo, Vilnius, Lithuania), three-layer cotton mask with antibacterial *Silverplus* coating (SIA P.E.M.T., Riga, Latvia) and a standard surgical mask type II EN 14,683 (Matopat, Toruń, Poland) were used as reference materials.

**Figure 1:**
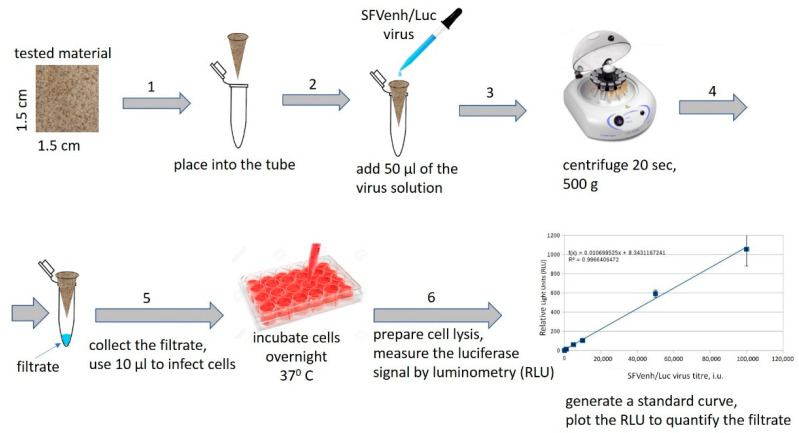
Schematic representation of the virus filtration test (technical details are provided in text). (1) 1.5 cm × 1.5 cm filter sample is cut out from each material, folded into the form of a conical funnel, and placed into a 1.5 mL tube; (2) 50 µL of recombinant Semliki forest virus (SFV)-enh/Luc virus solution (107 i.u./mL) is added into the cone; (3) the tube is centrifuged to allow the virus to pass through the material; (4) the filtrate (indicated by arrow) is collected; (5) the filtrated is diluted and used for cell infection in a 24-well cell culture plate; (6) after overnight incubation of the plate the cell lysates are prepared and the virus infection is measured by detection of the luciferase activity in infected cells (luminometry). The cell infection with the standard dilutions of the virus is used to generate a standard curve and to calculate the amount of virus in the filtrate.

A 1.5 × 1.5 cm sample was cut out from each material, folded in a form of a conical funnel, placed into truncated 1 mL plastic pipette tip (for better fixation), and subsequently placed into an Eppendorf 1.5 mL tube as presented in Figure 1.

50 µL of recombinant SFVenh/Luc virus solution with a titer of 10^7^ infectious units per milliliter (i.u./mL) were poured into each cone containing the test material. The tubes were then centrifuged for 20 s at 500× *g* (3500 rpm) by FVL-2400N Combi-Spin, Mini-Centrifuge/Vortex (Biosan, Riga, Latvia) to allow the liquid to pass (filtrate) through the material under low pressure conditions. The filtrated virus samples were collected (at least 20 µL each) and used for BHK-21 cell infection in 24-well plate as previously described [18]. Briefly, 10 µL of the virus sample in duplicate was mixed with 190 µL PBS (containing M^2+^ and Ca^2+^) and incubated with BHK-21 cells for 1 h at 37 °C, 5% CO_2_, then 800 µL of BHK medium (1% fetal bovine serum) was added. In parallel, standard dilutions of the SFVenh/Luc virus in a range 1 × 10^3^–5 × 10^5^ i.u./mL per well were generated and used for BHK-21 cell infection in duplicate in the 24-well plate. The cells were incubated overnight at 37 °C, 5% CO_2_ to allow complete cell infection to complete and expression of firefly luciferase gene.

To quantify the virus titer in filtered samples the relative luminescence units (RLU) were measured in cell lysates and the respective values were plotted on the standard curve with serial SFVenh/Luc virus dilutions. The RLUs were measured by the Luciferase assay (Promega, Madison, WI, USA), as recommended by manufacturer. Briefly, the cell medium was removed, and the cells (24-well) were lysed in 100 μL of the Cell Culture Lysis buffer (Promega, Madison, WI, USA), centrifuged at 600 cfr for 5 min, and 1 μL of the cell lysate was used immediately to measure the luciferase enzymatic activity by luminometer Luminoskan Ascent (Thermo Scientific, Loughborough, UK). The cell infection was done in duplicate in each independent experiment. Two independent experiments were performed with each sample (repeats). The virus titer standard curve was generated in each experiment and the negative control signal (RLU of uninfected cells) was subtracted from all values. The log_10_ reduction value (LRV) represents the difference between loaded and eluted virus infectious units per ml, and respectively was calculated according to the following equation:

LRV = [log_10_ (virus titer before filtration) − log_10_ (virus titer after filtration)]. (1)

Statistical analysis of obtained results was performed using an Excel 2016 MSO data statistical analysis tool.

## 3. Results and Discussion

The fruiting bodies of *L. lepideus*, *P. squamosus*, and *F. betulina* formed gelatinous biomass in NaOH solution which was unusable for further experiments. FF were successfully separated from the biomass of fungi *G. applanatum*, *F. fomentarius*, *A. bisporus*, and *T. versicolor* and integrated in biopolymer compositions with softwood and hemp cellulose fibers.

### 3.1. Morphological Characterization

**Figure 2:**
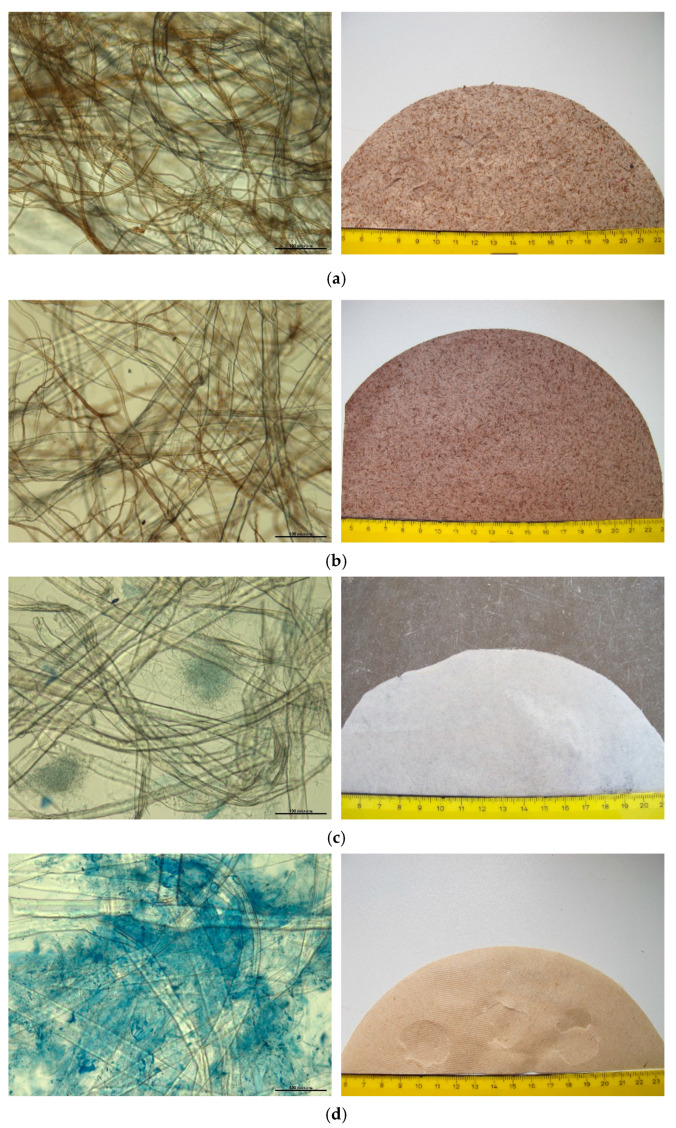

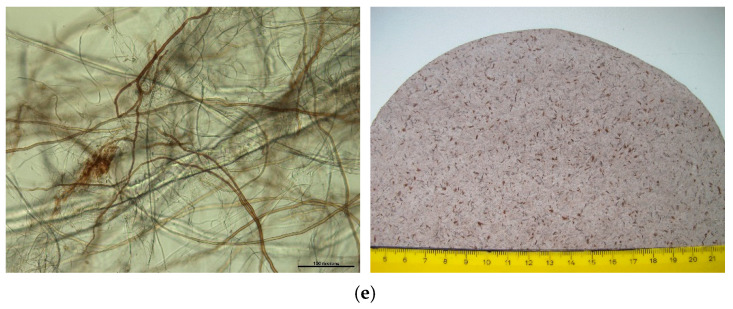
Microstructure (left column) and macrostructure (right column) of mycocel biopolymer compositions made of Kraft fibers (KF), hemp fibers (HF), and fungal fibers (FF): (**a**) KF FF (Ff); (**b**) KF FF (Ga); (**c**) KF FF (Tv); (**d**) KF FF (Ab); (**e**) KF HF FF (Ga). Microimages show flat hemp and softwood fibers (10–30 μm) (**a**–**e**) and narrow fungal fibers (2–7 μm) of polypores (**a**–**c**,**e**) and swollen hyphae (7–20 μm) of agaric (**d**). LM, 200×. Bar = 100 μm.

The microscopic structure of mycocel biopolymer blends and macrostructure of developed materials is shown in Figure 2.

Kraft fibers, hemp fibers, and fungal fibers were most likely physically bound together making a net of the biopolymer material. The fungal fibers separately or in bundles were randomly distributed within the net of cellulose fibers. The structure of materials was affected by individual properties of fibers. The size of Kraft fibers was average 2 mm in length and 30 μm in width. Hemp fibers were 1 mm long and 10–20 µm wide with a net of microfibers (2–5 μm). Fungal fibers from fruiting bodies of poroid species (*G. applanatum*, *F. fomentarius*) (Figure 2a,b) were 2–7 μm across. The fibers of *T. versicolor* mycelium were 2–3 μm wide (Figure 2c). The stipes of mushroom *A. bisporus* were composed of swollen hyphae, ascending 7–20 μm across (Figure 2d).

The hyphae of basidiomycetes (primarily poroid species) are divided in three main types: generative, binding, and skeletal hyphae with key differences in cell wall thickness, internal structure, and branching characteristics. Monomitic species comprise only generative hyphae, dimitic species comprise two hyphal types (usually generative and skeletal) and trimitic species contain all three hyphal types [21].

The fungal fruiting bodies under this study, namely, *G. applanatum* and *F. fomentarius*, had trimitic hyphal system. Depending on the species, the generative hyphae were 2–5 μm, skeletal hyphae 3–7 μm and binding hyphae 2–4 μm across (Figure 2a,b). In the case of poroid species *T. versicolor*, the mycelium of pure culture strain consisted of thin-walled generative hyphae, while hyphae of agaric *A. bisporus* were flat and swollen. The fibers of two later species formed dense hyphal bundles among the cellulose fibers. These can be observed in Figure 2c,d where cyanophilic reaction turned hyaline hyphae of Tv and Ab blue after staining. The trimitic species Ga and Ff presented brown pigmented hyphal network (Figure 2a,b,e). The pigmented layer within the hyphal cell wall is likely cross-linked to polysaccharides and has a mesh-like structure with pores, through which small and large molecules can penetrate into cells. Pigment melanin is believed to enhance the strength of the cell wall and has antioxidant properties and propensity to bind to a variety of substances [22].

**Figure 3:**
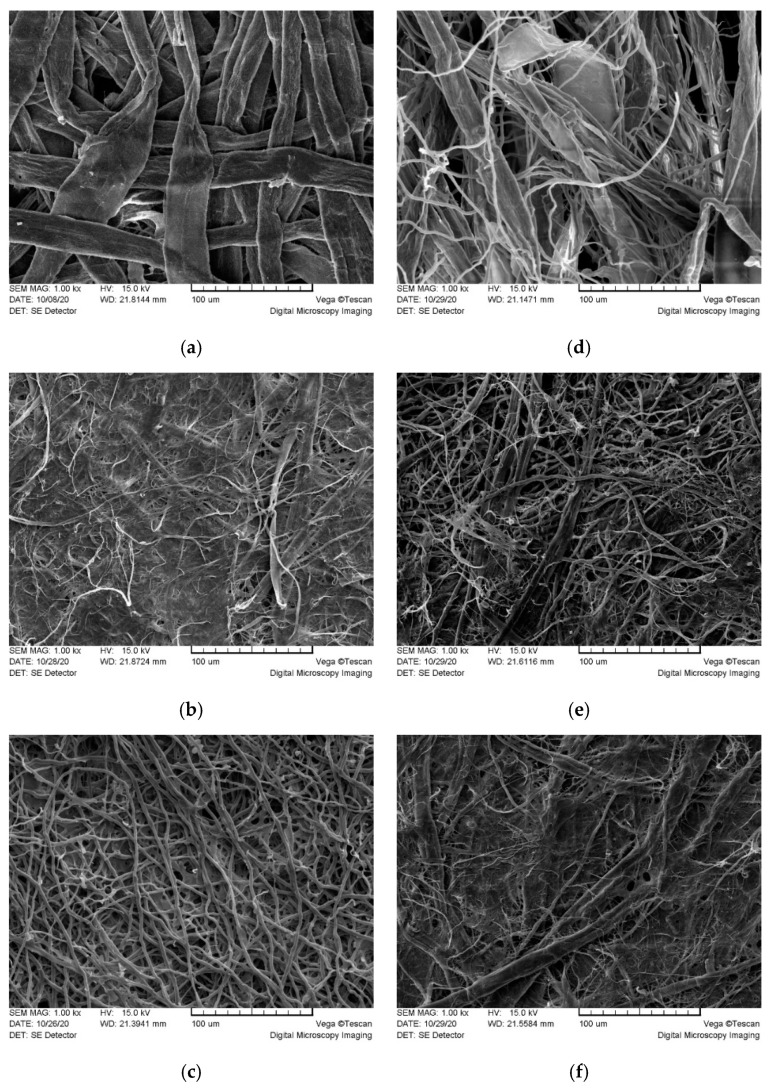
Ultrastructure of raw materials (left column) from (**a**) Kraft fibers (KF), (**b**) hemp fibers (HF), and (**c**) fungal fibers (FF Ga), and mycocel biopolymer compositions (right column): (**d**) KF FF (Ga); (**e**) HF FF (Ga); (**f**) KF HF FF (Ga). SEM, 1000×. Bar = 100 μm.

SEM micrographs (Figure 3) support the findings of LM and display a detailed ultrastructure of the raw materials and mycocel biopolymer blends. Fiber orientation appeared randomly distributed, entangled fibers were physically bound together with H bonds, forming non-oriented, multi-layered net. Kraft fibers (Figure 3a) were flattened and made a microporous network. Similarly, the fungal fibers Ga (Figure 3c) formed a network of microporous structures. Hemp material (Figure 3b) displayed a dense structure composed of flat fibers and thin microfibers.

The microstructure of mycocel biopolymers was affected by individual properties of raw materials. The structure of KF FF (Ga) (Figure 3d) was determined by individual properties of Kraft fibers and fungal fibers. Distribution FF among the flattened KF favoured formation a loose microporous structure of the material. The structure of biopolymer HF FF (Ga) was affected by a dense network of variable diameter hemp fibers (Figure 3e). The FF were incorporated in a tight HF network of microfibers forming a compact structure with decreased porosity. The surface of KF HF FF (Ga) material (Figure 3f) displayed dense areas of hemp fibers and Kraft fibers with few areas of loose fungal fibers. The porosity and the tortuosity fractal dimension are two critical parameters to determine the permeability. It is reported [23] that increase in the tortuosity fractal dimension leads to decrease in the dimensionless permeability and absolute permeability; an increase in the porosity increases the dimensionless permeability; increase in the fiber diameter yields an increase in the absolute permeability.

### 3.2. Air Permeability

The air permeability results were supported by microscopy findings. Mycocel biopolymer compositions had significant variations in air permeability properties starting from almost non-air permeable ones to materials with air permeability above measuring limit of device, which was 8820 mL/min (Table 1). The fungal type of hyphae had a significant effect on results. Biopolymer blends having *F. fomentarius* (Ff) or *G. applanatum* (Ga) trimitic hyphal system in combination with Kraft fibers (KF FF (Ff) and KF FF (Ga)) had the highest air permeability >8820 mL/min. Biopolymers consisting of cellulose fibers (KF and HF) and *A. bisporus* (Ab) hyphae had the lowest air permeability 14.1 mL/min, <1 mL/min and 7.5 mL/min for KF FF (Ab), HF FF (Ab), and KF HF FF (Ab), respectively.

It should be noted that materials with HF in their composition had the lowest air permeability numbers because of high Shopper Riegler freeness of hemp cellulose fiber (91.5 °SR) [12], which led to high bonding and tight networks of fibers (Figure 3b). The bonding of cellulose fibers is based primarily on hydrogen bonding between hydroxyl functional groups during close contacting of fibers [24]. The same bonding theory can be attributed to investigated mycocel biopolymer materials, since cellulose and chitin—the main constituent of hyphae—are biopolymers and have similar polysaccharide chain structure with the main difference being the replacement of one of the three hydroxyl groups with acetyl amine group in chitin monomeric unit [25]. The ability of functional groups of hyphal polysaccharides to make hydrogen bonding with cellulose fibers is one of the key elements of network formation in investigated mycocel materials. Furthermore, better bonding leads to a more compact packing of fibers and lower free volumes associated with lower air permeability [26], which can be seen in the cases of biopolymer blends with Tv mycelial and Ab stipe fibers. The higher numbers of air permeability showed lower ability of Ga and Ff hyphae to get involved in the network of cellulose fibers through hydrogen bonding. It can be explained by presence of non-polysaccharidic substances, such as pigments, which were indicated by the dark color of fruiting bodies and isolated hyphae from Ga and Ff.

Mycocel blends with higher air permeability have potential for using as gas permeable membranes, for example, as biobased filter layer in face masks. Disposal medical face masks, used as reference material, correspondingly showed high air permeability above 8820 mL/min, therefore only KF FF (Ga) and KF FF (Ff) are appropriate for this application. Blends with very low air permeability, such as those containing Ab hyphae can be used in applications, where high air or gas barrier properties are important, for example, food packaging materials [27].

### 3.3. Mechanical Properties

Comparison of cellulose and fungal fiber biopolymers showed significant differences among different fungal species and hyphal types regarding their effects on mechanical properties of investigated materials (Table 1). It was possible to produce a pure fiber material from *G. applanatum* (Ga) and *A. bisporus* (Ab) biomass, however FF (Ga) material had a rather low tensile index 8.2 Nm/g, but FF (Ab) material was too fragile for handling and it was not possible to measure mechanical properties. When compare two-component blends of Kraft fibers and fungal fibers, the best results were obtained in the case of KF FF (Ab) and KF FF (Tv), 32.5 and 26.5 Nm/g, respectively, which is by 92% and 57% more than tensile index measured for material containing only KF. However, addition of *G. applanatum* (Ga) or *F. fomentarius* (Ff) fibers to cellulose fibers decreased mechanical strength by 18% for KF FF (Ga) and by 48% for KF FF (Ff) if compare with pure KF. This can be explained by specific character of Ab and Tv hyphae which formed a dense microstructure with cellulose fibers increasing the mechanical strength of material. It is reported [28] that generative hyphae alone (monomitic hyphal system), which are hollow and contain cytoplasm, are suggested to provide limited mechanical performance, with binding hyphae (dimitic and trimitic hyphal systems) responsible for material strength. Contrary, our results showed that the trimitic hyphal system of fungi Ga and Ff did not favor an improvement of mechanical properties of mycocel compositions. Lower mechanical performance of Ga and Ff hyphae containing materials can be explained also by the aspects mentioned in description of air permeability properties and are related to lower ability for bonding with cellulose caused by presence of non-polysaccharidic substances and lower amount of hydrogen bonds formed. It is known that fiber to fiber bonding or bonding strength is directly related to mechanical strength of cellulose fiber-based materials and can be evaluated using results of mechanical tests [29].

Tensile index was almost threefold higher in wet stage when compare KF and KF FF (Ab) showing the ability of Ab hyphae to significantly improve the strength of fiber material in wet conditions. Two-component blends consisting of hemp fibers and fungal fibers HF FF were produced using hemp cellulose and Ga or Ab fibers. It must be noted that highly fibrillated hemp fibers with freeness 91.5 °SR used in the research, had the most significant effect on the mechanical strength of all investigated materials. Furthermore, pure HF material had the highest tensile index among all the tested materials by reaching 60.4 Nm/g. Addition of hyphae significantly decreased tensile index reaching 30.8 Nm/g in the case of HF FF (Ga) and 32.5 Nm/g in the case of HF FF (Ab). When compare three-component blends KF HF FF, here also Ab hyphae showed the highest effect on mechanical properties of composites by KF HF FF (Ab) reaching tensile index 46 Nm/g, while KF HF FF (Ga) reached 35.9 Nm/g. Overall, *A. bisporus* (Ab) and *T. versicolor* (Tv) fibers showed higher potential as component of biopolymer for improving the mechanical properties of cellulose fiber-based materials, whether they consist of wood or hemp cellulose fibers. Biopolymers containing Ga hyphae demonstrated the highest flexibility measured as percentage elongation or stretch. HF FF (Ga) and KF HF FF (Ga) samples reached 3.4% and 3.9% stretch, while other composites had numbers equal or below 2.0%.

Mechanical performance of materials was evaluated also by measuring burst index, which is the hydrostatic pressure necessary to cause rupture in a circular area of a given diameter and tells how much pressure material can tolerate before rupture. Three component blends showed the highest burst index 2.8 kPa m^2^/g and 2.4 kPa m^2^/g for KF FF FF (Ga) and KF HF FF (Ab) respectively, indicating the prevalence of fiber diversity presented in mechanical blend. If two-component blends are compared, Tv and Ab containing KF FF materials have higher results than Ga and Ff containing ones. Breaking length is used to characterize inherent strength of material and is defined as the length of imaginary material strip, if suspended vertically from one end, would break by its own weight. Numeric values of breaking length of biopolymer blends were in direct correlation with tensile strength values showing the predominance of Ab and Tv containing materials and lower results in Ga and Ff containing materials. Mechanical properties of disposal medical face masks layers were not possible to measure according to the standard and device used for biopolymer blends testing, however apparently they are less strong than materials of biopolymer blends.

### 3.4. Virus Filtration Properties

In order to evaluate the filtration properties of the experimental biomaterials, the recombinant Semliki forest virus (SFV) was applied, which belongs to the Togaviridae family of enveloped RNA viruses (60–70 nm in diameter) structurally similar to human pathogenic viruses such as influenza and coronaviruses. The test system was based on a safe replication deficient SFV vector (pSFVenh/Luc), allowing to perform one round cell infection to be performed with precise quantification of the virus filtration rates using rapid measurement of luciferase activity in infected cells.

The efficiency of the SFV/enhLuc virus filtration through the tested materials are summarized in Table 2. The efficient virus retention properties were observed both for raw materials (Kraft fibers, hemp fibers, and fungal fibers Ga) and mycocel blends KF FF (Ga) and KF FF (Tv), which revealed very low virus permeability rates (<2%). Cellulose hygiene paper also showed the ability to retain the virus, albeit with lower efficiency (the permeability <30%) comparing to cellulose containing biopolymers. The properties of cellulose materials are determined by production technology and additives which can result in lower mechanical strength, density, and virus filtering efficiency as observed in the case of hygiene paper. Regarding the reference materials, i.e., face masks, the highest virus permeability was observed for the tested surgical mask (>92%) and hydrophobic outer layer of the cotton mask (>70%), whereas the *Silverplus* layer retained the virus significantly (the permeability <1%).

According to EMA guidelines (CPMP/BWP/268/95, European Medicines Agency) the log_10_ reduction value (LRV) is an important parameter to quantify the virus reduction capacity. Only the LRV > 4 is considered as a “very high” virus reduction potential. In this study, the biopolymer KF FF (Ga) demonstrated sufficiently high virus reduction capacity (4.54 LRV). Remarkably, the silver containing cotton layer of the commercial mask also showed efficient virus removal properties (3.6 LRV), which can be related to the viricidal capacity of the immobilized silver nanoparticles [30].

Due to safety aspects, handling of human viruses is a labor-intensive and time-consuming process. In this study, we have established a protocol for a relatively simple virus filtration method, which is based on replication deficient SFV vector encoding firefly luciferase used for rapid virus quantification. The proposed method can be efficiently applied for primary screening of virus filtration properties of the membranes.

The mechanisms underlying the membrane filtration of viruses include size exclusion and/or adsorptive interactions (e.g., hydrophobic/hydrophilic and electrostatic interactions) between virus envelope and membrane compounds [31]. The diameter of the virus particles is much smaller than the pore size of the tested membranes. Therefore, the adsorptive interactions can be considered as the main mechanism of filtration in the tested system. A surgical mask, which is made of hydrophobic polypropylene layers, did not adsorb aqueous virus containing solution, resulting in low virus retention values (Table 2). Practically, the surgical masks serve as a barrier to aqueous aerosol and are designed to block direct fluid entry into the wearer’s respiratory tract and mostly act as a repellent of the water-based liquids [32]. In contrast to surgical mask and tested cotton mask, the row fiber materials and mycocel blends exhibited hygroscopic properties and showed the ability to binding, or adsorption of SFV particles. A high virus filtration capacity of mycocel biopolymer blends might be attributed to the structural properties of the fungal cell wall as an insoluble polysaccharide-based sorbent. The fungal polysaccharides possess direct virus inactivation properties as shown for several highly pathogenic human viruses, including human immunodeficiency virus, herpes simplex virus, etc. [33]. The application of mycocel-based filters alone or in combination with polypropylene-based layers can represent an advanced individual respiratory protective device against airborne pathogens. Furthermore, chitin, one of the main polymers of fungal cell walls, is widely used for controlled drug delivery systems, protein and enzyme carriers, and packaging materials, based on its natural antimicrobial activity. Chitin and its derivatives (e.g., chitosan) have many useful properties that make them suitable for a wide variety of biomedical applications. Their products are known to be antibacterial, antifungal, antiviral, nontoxic, and nonallergic [34]. Therefore, the proposed mycocel-based biopolymers are promising multifunctional materials for biomedical and bioengineering applications.

## 4. Conclusions

A novel mycocel biopolymer from naturally derived biomass of plant cellulose fibers and fungal fibers (hyphae) was developed and characterized regarding its air permeability, mechanical properties, and virus filtration efficiency.

Air permeability and mechanical properties of mycocel biopolymer blends were affected by microstructural features of raw materials. Highly fibrillated hemp fibers had the most significant effect on the mechanical strength while Kraft fibers revealed increased air permeability. The ability of functional groups of hyphal polysaccharides to make hydrogen bonding with cellulose fibers was one of the key elements of network formation which determined biopolymer properties. The loose fiber net of trimitic fungal species *G. applanatum* and *F. fomentarius* with incorporated Kraft fibers formed a microporous structure of mycocel blends with improved air permeability (>8820 m/min) and limited mechanical properties in comparison with individual raw fibers. *A. bisporus* and *T. versicolor* fibers showed higher potential as components of biopolymer for improving the mechanical properties of cellulose fiber-based materials, whether they consist of wood or hemp cellulose fibers.

Virus testing provided promising results regarding virus filtration efficiency of biopolymer blends. Mycocel biopolymer KF FF (Ga) demonstrated sufficiently high virus reduction capacity (4.54 LRV) than surgical mask and outer and inner layers of commercial face mask. Feasibly, the adsorptive interactions can be considered as the main mechanism of filtration in tested system.

The natural, biodegradable mycocel blends have a potential for use in biomaterial membranes depending on the target application. Blends with higher air permeability and virus filtration efficiency have potential for being used as gas permeable membranes, for example, as biobased filter layer in face masks. Blends with low air permeability can be used in areas where high air or gas barrier properties are important, for example, food packaging materials. The water microfiltration and ultrafiltration also are considered for future application.

## Figures and Tables

**Table 1:** Mechanical and air permeability properties of mycocel biopolymer materials (KF = Kraft fibers; HF = hemp fibers; FF = fungal fibers; Ga = *G. applanatum*; Ab = *A. bisporus*; Tv = *T. versicolor*; Ff = *F. fomentarius*). The marked (*) samples have been described previously [12].

Sample	Tensile Index, Dry Nm/g	Tensile Index, Wet Nm/g	Burst Index kPa m^2^/g	Breaking Length km	Stretch %	Air Permeability mL/min
KF *	16.9	0.8	1.0	1.7	0.9	8275
HF *	60.4	10.9	4.6	6.1	3.4	32
FF (Ga) *	8.2	-	0.9	0.8	2.0	6935
KF FF (Ga) *	13.9	1.3	1.0	1.4	2.0	>8820
HF FF (Ga) *	30.8	-	2.1	3.1	3.4	77
HF FF (Ab)	32.5	5.3	2.0	3.3	0.7	<1
KF HF FF (Ga) *	35.9	1.9	2.8	3.7	3.9	115
KF FF (Tv)	26.5	2.4	2.0	2.7	2.0	1630
KF FF (Ff)	8.9	-	1.5	0.9	1.1	>8820
KF FF (Ab)	43.5	3.0	1.9	4.4	1.0	14.1
KF HF FF (Ab)	46.0	3.0	2.4	4.7	1.6	7.5

**Table 2:** SFV1enh/Luc recombinant virus titer change after pressure filtration through experimental materials (KF = Kraft fibers; HF = hemp fibers; FF = fungal fibers; Ga = *G. applanatum*; Tv = *T. versicolor*).

Sample	Virus Titer i.u./mL ± SD	Virus Titer [Log_10_]	Log_10_ Reduction Value LRV	Virus Amount after Filtration Relative to Nonfiltered Control %
Non-filtered virus	(1 ± 0.066) × 10^7^	7.00	-	100
Surgical mask (all layers)	(8.28 ± 0.066) × 10^6^	6.92	0.08	92.63
Cellulose hygienic paper	(2.66 ± 0.076) × 10^6^	6.42	0.58	27.05
KF	(1.43 ± 0.413) × 10^4^	4.16	2.84	0.16
KF FF (Ga)	(2.86 ± 0.076) × 10^2^	2.46	4.54	0.00
KF FF (Tv)	(7.66 ± 0.791) × 10^4^	4.88	2.12	0.78
FF (Ga)	(2.25 ± 1.460) × 10^4^	4.35	2.65	0.26
HF	(6.27 ± 2.120) × 10^3^	3.80	3.20	0.08
Cotton outer layer (hydrophobic)	(7.21 ± 0.0330) × 10^6^	6.86	0.14	73.44
Cotton Silverplus middle layer	(2.52 ± 3.350) × 10^3^	3.40	3.60	0.03
Cotton inner layer	(7.31 ± 1.300) × 10^5^	5.86	1.14	7.44

## Data Availability

Not applicable.
